# Nature and Treatment of Vaginismus

**Published:** 1873-07

**Authors:** 

**Affiliations:** St. Catharines


*Nature and Treatment of Vaginismus.* By Dr. Mack, of St. Catharines. Read before the Medical Society, St. Catharines.

This extremely annoying disease is so perfectly under the control of the surgeon, that it should be placed among the well recognized forms of complaint, for which his resources are called upon. Efforts have been made of late to call in question the propriety of Sims' or Simpson's operations, based doubtless upon cases of abuse.

Having operated in all, about ten times, I would now submit a condensed history of two typical examples of the disease and its treatment.

Mrs. G., married eighteen months, menstruation regular and normal except in being attended with some dorsal and hypogastric pain. Sexual intercourse extremely unpleasant and painful, yet tolerated, although with much repugnance; her husband complained to Dr. M. that his married life had been very far indeed from what he had expected it to be. Examination per vaginam proved to be very difficult from the great sensitiveness of the ostium vaginæ; examination by the speculum was not to be attempted without anæsthesia. The carunculæ myrtiformes and remains of the hymen were florid and large, but the seat of most exalted sensation appeared to be at the fourchette, accompanied by spasm of the sphincter vaginæ. Examination under chloroform showed symptoms of endo-cervicitis.

The following day, full insensibility having been produced, I introduced two fingers of my left hand, and having divaricated them so as to put the parts fully on the stretch, I made two incisions so that they should represent the letter Y, the oblique part of the incisions commencing about two inches up the vagina, about one inch from the mesial line along the posterior wall, dividing the mucous coat, and after being continued to within less than one inch from the perineal surface, then carried straight in the mesial line, and dividing the soft parts freely for about half an inch from the raphe perinei the same oblique portion of the incision was then made upon the opposite side.

Pledgets of charpie soaked in a weak solution of persulphate of iron were placed in the incisions, and a compress secured by a T bandage; the strictest quiet and rest in bed were enjoined. Cold applications of lint, wet with iced water, were kept up, the bladder being relieved every eight hours by using the catheter. After forty-eight hours the dressings were removed, and one of Sims' glass dilators directed to be introduced occasionally, and retained for about two hours.

Four days after the operation, the use of the dilators proving extremely painful, full anæsthesia having been induced again, the remains of the hymen were carefully and thoroughly removed by a small curved scissors. No hemorrhage followed, and in about twenty-four hours the dilators could be tolerated. Two weeks after the first operation the local treatment for the inflammatory condition of the cervix uteri was commenced, and after about two months treatment a complete cure was effected, resulting in the birth of a healthy son in less than ten months. This case is one of the milder type; the next is a fair sample of the more severe forms of the disease.

Mrs. M., married about three years, without issue, declares that perfect connection has never been effected. Her husband has been morbid and unhappy, and the matrimonial alliance is likely to terminate in great misery for all parties. Examination per vaginam could not be thought of, the attempt was violently repelled by involuntary struggles. After inhalation of chloroform, examination disclosed the hymen partially ruptured, and the entrance of the vagina rigid, and small as in the virgin state; keeping up the anæsthesia the incisions were at once made as already described, and then immediately the remains of the hymen were carefully dissected away. Persulphate of iron was applied to the bleeding surfaces, and a small glass dilator was introduced and maintained by compress and bandage.

In twenty-four hours the dilator was removed, wet compresses were applied, and forty-eight hours after the operation a larger dilator was introduced for about twelve hours. From this time, for about fourteen days, the dilators were inserted, gradually increasing their size at intervals of about two hours, and retained about two hours each period. A speculum was then introduced, and local treatment directed to a slight endo-cervicitis, which yielded in the space of six or seven weeks.

The lady returned to New York, her residence, and in less than one year from that time, she gave birth to a fine healthy infant.

The first report of any treatment for this distressing neurosis is to be found in Burns' Principles of Midwifery, where, in the portion of his book assigned to describing the anatomy of the pelvis, and when treating of the pudic nerve, it is stated: "The pudic nerve, after re-entering the pelvis, gives off several small branches, which go to the obturator internus, sphincter ani, and extremity of the rectum. It then divides into two. The trunk as it may be called, runs forward with the artery to the clitoris, covered as it proceeds along the rami of the pubis, by the erector.

The other division is distributed to the perineum and vagina. It approaches the vagina nearly in a line with its junction with the perineum, and subdivides and ramifies on the end of that passage, but chiefly on its orifice.

This nerve is often preternaturally sensible, so as to cause great pain in coition as well as at other times. It may be exposed by cutting through the skin and fascia, at this side of the labium and perineum beginning in a line with the front of the vaginal orifice, and carrying the incision back for two inches. The nerve being blended with cellular substance, is not easily seen in such an operation, but it may be divided by turning the blade of the knife and cutting through the vagina to its inner coat, but not injuring that. It may be more easily divided by cutting from the vagina. Slitting merely the orifice of the vagina will not do. We must carry the incision fully half an inch up from the orifice of the vagina, and also divide the mucous membrane freely in a lateral direction.

In another place he tells us that the sensitiveness is sometimes dependent on little tubercles or inflamed patches at the orifice, in which case, we may try the free application of nitrate of silver with or without scarification.

But if there be no tubercles, and especially if there be tightness at or within the orifice, we must in one or more places divide the mucous coat, as high as there is anything like a band.

Dr. Neftel reports a case successfully treated by electricity.

Dr. Mahend also, in his book "*Sterilite chez la Femme*," reports a success from the same mode of treatment.

There are other cases of pain in coition, distinct from vaginismus which must be borne in mind, *e.g.*, painful affections of any of the parts adjacent to or contained in the vulva, inflammation of Bartholine's follicles.

The operation performed by me depends, I believe, wholly for success upon the after treatment, by keeping up dilatation, for all of which we are indebted to the practical ingenuity of Dr. Marion Sims.

I wish to be clearly understood that I do not advocate the performance of this operation in all cases of vaginodynia indiscriminately, but in cases where it is not due to hysteria curable by constitutional measures, or to fissures and sores of the vulva, eruptions or neuromata, vaginitis or metritis in any of its forms capable of treatment, locally or generally, or to tubercle of the meatus urinarius, but rather to those cases dependent upon spasm of the sphincter vaginæ with an excessively irritable condition of the nervous filaments. Emmett divides the fibres of the sphincter, and the tense corded band usually to be found at some part of the vaginal wall.

I do not think that vaginismus depends wholly upon spasm of the sphincter vaginæ, but upon pain in the faciæ and muscles deriving sensation from the branches of the pudic nerve and which must, of necessity, be divided and kept from re-uniting by the method of Sims.

Debout, Chamie, and Muhon, all recommend the operation as described by Burns. Simpson operated subcutaneously with a tenotome.

Menville de Pouseen recommends, when the affection will not yield to constitutional and local mild remedies, cauterization of the inferior portion of the vaginal orifice. Lisfranc reports a case cured by bougie. I have seen belladonna, atropine and the glass dilators succeed, but in our devotion to conservative surgery, we must draw the distinction between that and no surgery, and remember that the cruelty lies in losing time and creating suffering from months of futile efforts at gradual or forcible dilatation, or wearing out the patience of all concerned with quantities of medicaments consigned to the stomach, local baths, poultices or ointments.

Pregnancy would prove a sure remedy, but I think it likely that the indelicate proceeding of anæsthetizing a woman and leaving her to the marital embrace, as reported by another practitioner, would prove very difficult to reduce to general practice.

In conclusion, I would state that, with one exception, the cases I have met with were found among that class of society in which the intellectual faculties are too often exercised at the expense or neglect of the physical.—*Canada Lancet.*

